# The importance of patient-centered care and co-creation of care for satisfaction with care and physical and social well-being of patients with multi-morbidity in the primary care setting

**DOI:** 10.1186/s12913-018-3818-y

**Published:** 2019-01-08

**Authors:** Sanne Jannick Kuipers, Jane Murray Cramm, Anna Petra Nieboer

**Affiliations:** Erasmus School of Health Policy & Management, Erasmus University Rotterdam, Rotterdam, the Netherlands

**Keywords:** Patient-centered care, Co-creation of care, Multi-morbidity, Primary care, Physical well-being, Social well-being, Satisfaction with care

## Abstract

**Background:**

Patients with multi-morbidity have complex care needs that often make healthcare delivery difficult and costly to manage. Current healthcare delivery is not tailored to the needs of patients with multi-morbidity, although multi-morbidity poses a heavy burden on patients and is related to adverse outcomes. Patient-centered care and co-creation of care are expected to improve outcomes, but the relationships among patient-centered care, co-creation of care, physical well-being, social well-being, and satisfaction with care among patients with multi-morbidity are not known.

**Methods:**

In 2017, a cross-sectional survey was conducted among 216 (of 394 eligible participants; 55% response rate) patients with multi-morbidity from eight primary care practices in Noord-Brabant, the Netherlands. Correlation and regression analyses were performed to identify relationships among patient-centered care, co-creation of care, physical well-being, social well-being, and satisfaction with care.

**Results:**

The mean age of the patients was 74.46 ± 10.64 (range, 47–94) years. Less than half (40.8%) of the patients were male, 43.3% were single, and 39.3% were less educated. Patient-centered care and co-creation of care were correlated significantly with patients’ physical well-being, social well-being, and satisfaction with care (all *p* ≤ 0.001). Patient-centered care was associated with social well-being (*B* = 0.387, *p* ≤ 0.001), physical well-being (*B* = 0.368, *p* ≤ 0.001) and satisfaction with care (*B* = 0.425, *p* ≤ 0.001). Co-creation of care was associated with social well-being (*B* = 0.112, *p* = 0.006) and satisfaction with care (*B* = 0.119, *p* = 0.007).

**Conclusions:**

Patient-centered care and co-creation of care were associated positively with satisfaction with care and the physical and social well-being of patients with multi-morbidity in the primary care setting. Making care more tailored to the needs of patients with multi-morbidity by paying attention to patient-centered care and co-creation of care may contribute to better outcomes.

**Electronic supplementary material:**

The online version of this article (10.1186/s12913-018-3818-y) contains supplementary material, which is available to authorized users.

## Background

Because of aging populations, the prevalence of multi-morbidity has grown tremendously and is expected to increase even further in the near future [1, 2]. This increase poses a challenge, as patients with multi-morbidity have complex care needs that often make adequate healthcare delivery difficult and costly to manage [3]. Most current healthcare systems are single disease–oriented and thus not adequately responsive to patients with multiple diseases and combinations of complex care needs. Healthcare for patients with multi-morbidity involves following multiple disease-specific guidelines that do not take aspects of multi-morbidity into account, resulting in a deficiency of evidence regarding best treatment [4, 5]. Current care delivery is not tailored to the needs of patients with multi-morbidity [6], despite the heavy burden that multi-morbidity places on these patients. This burden is often related to adverse patient outcomes, leading to a greater risk of mortality and increased healthcare utilization and cost [7]. As a result, patients with multi-morbidity report lower quality of life and well-being, and less satisfaction with care [3, 8]. Making care more patient-centered may be the way forward.

Patient-centered care (PCC) has the potential to make care more tailored to the needs of patients with multi-morbidity. PCC can be defined as “providing care that is respectful of and responsive to individual patient preferences, needs, and values and ensuring that patient values guide all clinical decisions” [9]. Previous studies have investigated patients’ perspectives on PCC and distinguished eight dimensions: (1) patients’ preferences, (2) information and education, (3) access to care, (4) emotional support, (5) family and friends, (6) continuity and transition, (7) physical comfort, and (8) coordination of care [10]. According to a systematic review conducted by Rathert and colleagues [11], organizations that are more patient-centered also have more positive outcomes, such as greater satisfaction with care, greater job satisfaction among healthcare professionals, increased quality and safety of care, and greater quality of life and well-being of patients. However, the systematic review included mainly studies conducted in hospital settings; very few were conducted in primary care settings and they did not specifically target patients with multi-morbidity. Although PCC is expected to be beneficial for patients with multi-morbidity, the relevance of its eight dimensions for these patients in the primary care setting is not known. Given that PCC may differ among settings [11], investigation of its effects on patients with multi-morbidity in the primary care setting is important.

### Co-creation of care

In addition to the eight dimensions of PCC, which inform us how patient-centered organizations are, examination of co-creation of care is important. Co-creation of care is based on the quality of relationships characterized by patient-centered interaction and communication, which is also important for improving outcomes [12, 13]. Co-creation of care is the establishment of productive interactions between patients and healthcare professionals [13]. Productive interactions are defined as timely, accurate, and problem-solving ways of communication [14]. According to Gittell [14], three relational dimensions are particularly important for establishing such productive interactions: shared goals, shared knowledge, and mutual respect. Co-creation is especially important in situations characterized by complex tasks, uncertainty, and time constraints. A meta-synthesis by Cottrell and Yardley [15] showed that patients, general practitioners (GPs), and medical interns experience the complexity of managing care for patients with multi-morbidity, and they face difficulties and uncertainties in finding the type of care necessary to meet all of these patients’ needs and wishes. Moreover, GPs find that care delivery to patients with multi-morbidity is often time consuming because of single-disease-oriented systems and their accompanying logistics. These difficult and complex issues thus make the co-creation of care potentially valuable in the context of care delivery to patients with multi-morbidity. Co-creation of care is expected to lead to better outcomes among these patients.

### PCC and patient outcomes

Physical and social well-being and satisfaction with care are important outcomes for patients with multi-morbidity [6]. Programs that improve the quality of primary care are associated with better outcomes, such as improved physical well-being, but are not able to prevent the decline in social well-being of patients with chronic illnesses [16]. Making chronic care more patient-centered is expected to enable patients to manage their own health and quality of life, thereby improving their physical and social well-being and satisfaction with care [16]. Rathert and colleagues [11] reported positive relationships between PCC and patients’ well-being and satisfaction with care, but their review did not include studies of patients with multi-morbidity in the primary care setting. The relationships among PCC, co-creation of care, patients’ well-being (physical and social), and patients’ satisfaction with care remain unexamined among patients with multi-morbidity.

### Study aim

Although we hypothesize positive associations among PCC, co-creation of care, physical and social well-being, and satisfaction with care among patients with multi-morbidity, research supporting these expectations is still lacking. Therefore, this study aimed to explore the current level of PCC delivery to patients with multi-morbidity in the primary care setting and the relationships among patient-centered care, co-creation of care, satisfaction with care, and physical and social well-being of patients with multi-morbidity.

## Methods

This study included multi-morbid patients from eight primary care practices in Noord-Brabant, the Netherlands. All patients with two or more registered chronic conditions (*n* = 413) were eligible to participate. Exclusion criteria were: too ill to participate or recently moved (and as a result no longer treated by the primary care practices under study). Based on information received from the GP, patient or their informal caregiver nineteen patients were not eligible to participate (death (*n* = 4), terminal illness (*n* = 2), incorrect address (*n* = 5), recent move (*n* = 2), inability to fill in the questionnaire due to poor cognitive functioning (*n* = 2), recent stroke (*n* = 1), or poor eyesight (*n* = 3)). Questionnaires were sent by mail to all remaining participants (*n* = 394). After a few weeks, reminders were sent to non-respondents. Another few weeks later, second reminders and duplicates of the questionnaire were sent to non-respondents. When no response was received after the second reminder, we called non-respondents for whom telephone numbers were available. In total, 216 patients filled in the questionnaire and consented to participate in the study. Thus, the response rate was 55% (216 out of 394 respondents). A sample size calculation revealed that 110 participants would be required in order to detect small to medium effects with 95% power and a type 1 error rate of 5% [17]. Having 216 respondents is therefore sufficient for valid results.

The medical ethics committee of the Erasmus Medical Centre, Rotterdam, the Netherlands, reviewed the research proposal (file number METC_2018_021) and decided that the rules laid down in the Dutch Medical Research Involving Human Subjects Act did not apply. Our research did not have a RCT design, participants were not subjected to procedures such as taking a blood sample, the research was not carried out with the intention of contributing to medical knowledge (e.g. etiology, pathogenesis, signs/symptoms, diagnosis) by systematically collecting and analyzing data. The main aim of the research was to investigate experiences of participants with care delivery, a process evaluation to improve quality of care delivery, which does not fall under the scope of Medical Research Involving Human Subjects Act (WMO) (see https://english.ccmo.nl/investigators/legal-framework-for-medical-scientific-research/your-research-is-it-subject-to-the-wmo-or-not). Written consent was obtained from all participants.

### Measures

#### PCC for patients with multi-morbidity in the primary care setting

PCC for patients with multi-morbidity in the primary care setting was measured using the 36-item patient-centered primary care (PCPC) instrument, which assesses the eight dimensions of PCC [18]. The PCPC instrument builds on our earlier work, in which we investigated the eight dimensions of PCC in hospital and long-term care settings [19–21]. Responses of patients were measured on a 5-point scale ranging from 1 (totally disagree) to 5 (totally agree), with higher scores indicating greater PCC. Scores for each of the eight dimensions of PCC were derived by calculating the average score for all items in that particular dimension. The overall score of PCC, in turn, was derived by calculating the average score for the eight dimensions (mean of the eight subscales calculated in the previous step). In this study, the Cronbach’s alpha value for this instrument was 0.89, indicating good reliability.

#### Well-being

Well-being was measured with the 15-item version of the Social Production Function Instrument for the Level of Well-being (SPF-ILs) [22]. Levels of physical (comfort and stimulation) and social (status, behavioral confirmation, and affection) well-being were measured. Responses of patients were measured on a 4-point scale ranging from 1 to 4, with higher scores indicating greater well-being. Scores for physical and social well-being were derived by calculating the average score for all items in that particular subsection of items. In this study, the Cronbach’s alpha value for both physical and social well-being, measured with the SPF-ILs, was 0.83, indicating good reliability.

#### Co-creation of care

Co-creation of care was measured with the relational co-production instrument [23]. The instrument consists of seven items measuring four aspects of communication (timely, accurate, frequent, and problem-solving) and three aspects of the relationship (shared goals, shared knowledge, and mutual respect) between patients with multi-morbidity and the healthcare professionals treating them (GPs, nurse practitioners, and specialists). Responses of patients were measured on a 5-point Likert-scale ranging from 1 (never) to 5 (always), with higher scores indicating better co-creation of care. Scores for co-creation of care were derived by calculating the average score for all items in this instrument. In this study, the Cronbach’s alpha value for this instrument was 0.93, indicating excellent reliability.

#### Satisfaction with care

The adjusted version of the Satisfaction with Stroke Care questionnaire (SASC) was used to measure patients’ satisfaction with care [24]. Although the original 8-item SASC was used among stroke patients, this instrument contains generic questions about satisfaction with care and is not restricted to patients receiving stroke care. The SASC instrument is therefore often used in various patient populations in the hospital setting [25–28]. Given that the instrument was developed to assess satisfaction with care in the hospital setting, we did slightly adjust items for the primary care setting (e.g. ‘The doctors have done everything they can to make me well again’ was changed into ‘The staff has done everything they can to make me well again’). Furthermore, we removed irrelevant or overlapping items (e.g. ‘The hospitalization process went smoothly’ and ‘I have been treated with kindness and respect by the staff at the hospital’), which resulted in a final set of 6 items: ‘I have received all the information I want about the causes and nature of my illness(es)’, ‘The staff has done everything they can to make me well again’, ‘I am satisfied with the type of treatment they have given me (e. g. physiotherapy, occupational therapy)’, ‘I have had enough therapy (e.g. physiotherapy, occupational therapy)’, ‘I am happy about the effect treatments had on my disease progression’, and ‘I am satisfied with the treatment provided by the general practitioner who I visit’. Responses of patients were measured on a 4-point scale ranging from 1 (totally disagree) to 4 (totally agree), with higher scores indicating greater satisfaction with care. Satisfaction with care scores were derived by calculating the average score for all 6 items. In this study, the Cronbach’s alpha value for this instrument was 0.89, indicating good reliability.

#### Background characteristics

Patients were also asked to provide information on background characteristics, such as age, gender, education, and marital status. Dummy variables were created for marital status (1, living alone, widowed or divorced; 0, married/living with partner) and education (1, primary education or less; 0, preparatory school for vocational secondary education or higher).

### Statistical analyses

SPSS software (version 23; IBM Corporation, Armonk, NY, USA) was used to analyze the data. Descriptive statistics were applied to all variables and involved the calculation of *n*s, means, minimums, maximums, standard deviations (SDs), and/or percentages. Pearson correlation analyses were performed to identify associations between PCC and background characteristics, co-creation of care, satisfaction with care, and physical and social well-being of patients with multi-morbidity. Regression analyses were performed to investigate multivariate relationships among these variables. Two-sided *p* values ≤0.05 were considered to be significant.

As data were missing for some PCC items due to occasional inapplicability, we additionally employed multiple imputation techniques (Markov chain Monte Carlo) and performed the regression analyses on pooled results based on the five imputed datasets (*n* = 216 each). Predictive mean matching was used as an imputation model to ensure that imputed values preserved the actual range of each variable.

## Results

Table 1 displays the background characteristics of the patients. Their mean age was 74.46 ± 10.64 (range, 47–94) years. Less than half (40.8%) of the patients were male, 43.3% were single, and 39.3% had low educational levels.

**Table 1:** Descriptive statistics (*n* = 216)

Characteristic	Mean ± standard deviation (range) or percentage
Age (years)	74.44 ± 10.64 (47–94)
Gender (male)	40.8%
Education (low)	39.3%
Marital status (single)	43.3%
Patient-centered care	3.84 ± 0.47 (1.7–5)
Preferences	3.96 ± 0.63 (1–5)
Physical comfort	3.92 ± 0.57 (1.8–5)
Coordination	3.92 ± 0.61 (2–5)
Emotional support	3.45 ± 0.75 (1–5)
Access to care	3.99 ± 0.56 (1.67–5)
Continuity and transition	3.97 ± 0.58 (2–5)
Information and education	3.89 ± 0.56 (2–5)
Family and friends	3.57 ± 1.01 (1–5)
Co-creation of care	3.61 ± 0.85 (1–5)
General practitioner	3.78 ± 0.88 (1–5)
Nurse practitioner	3.63 ± 1.03 (1–5)
Specialist	3.12 ± 1.32 (1–5)
Satisfaction with care	3.13 ± 0.45 (1.5–4)
Social well-being	2.71 ± 0.53 (1.44–4)
Physical well-being	2.55 ± 0.62 (1–4)

Note: Based on imputed data

The mean overall score for the level of PCC in the primary care practices was 3.84 ± 0.47. PCC dimension scores ranged from 3.45 (SD 0.75) to 3.99 (SD 0.56). The mean scores for the emotional support and family and friends dimensions were relatively low (3.45 and 3.57, respectively). The mean score for co-creation of care was 3.61 ± 0.85. GPs received the highest co-creation of care score (3.78 ± 0.88), followed by nurse practitioners (3.63 ± 1.03) and specialists (3.12 ± 1.32). The mean satisfaction with care score was 3.13 ± 0.45. The mean scores for social and physical well-being were 2.71 ± 0.53 and 2.55 ± 0.62, respectively; these scores were lower than those obtained among patients with chronic obstructive pulmonary disease (COPD), cardiovascular disease (CVD), and diabetes (see Additional file 1: Table S1).

Table 2 shows the percentage of patients who (completely) agreed with each PCC item (if applicable). About half of patients agreed with the items in the emotional support dimension. In the patient preferences dimension, about three-fourths of patients agreed with the items “I was helped to determine my own treatment goals,” “I felt supported to achieve my treatment goals,” and “I received advice that I really could use.” In the physical comfort dimension, 60% of patients felt that attention was given to fatigue and insomnia, 74.3% felt that the waiting rooms were comfortable, and 71.5% felt that they had sufficient privacy in the treatment room and at the counter. An important issue in the access to care dimension seems to be waiting time; slightly more than 30% of patients felt that they had been waiting too long to be seen by care providers. In the information and education dimension, about half of the patients felt that their own data was easily accessible. Finally, there is room for improvement in the friends and family dimension, especially concerning the items “attention was given to care and support provided by family members” and “attention was given to possible questions from my family members.” When applicable, more than one-third of respondents were dissatisfied about the way in which care providers involved family and friends.

**Table 2:** Percentages of respondents’ agreement with patient-centered care items

Patient-centered care item	(Completely) agree (%)
Patient preferences	
I felt taken seriously	89.2
My wishes and preferences were taken into account when choosing a treatment	80.4
I was involved in decisions about my treatment	85.7
The influence that the treatment can have on my life was taken into account	80.4
I was helped to determine my own treatment goals	73.5
I felt supported to achieve my treatment goals	77.0
I received advice that I really could use	79.0
Physical comfort	
Attention was given to my physical comfort (such as the management of pain, shortness of breath)	84.7*
Attention was paid to fatigue and insomnia	60.8*
The (waiting) rooms were clean	90.0
The (waiting) rooms were comfortable	74.3
In the treatment room(s) and at the counter there was sufficient privacy	71.5
Coordination of care	
Everyone was well informed; I only had to tell my story once	81.5*
The care was well attuned among the practitioners involved	81.7*
I knew who was coordinating my care	71.9
I could easily contact someone with questions	79.4
Continuity and transition	
When being referred to another care provider (specialist/dietician/physiotherapist) I was well informed about where to go and why	86.0*
With a referral, all my information was passed on correctly	82.2*
Advice (such as medication) from different practitioners (medical specialists and family doctor) was well attuned to each other	78.7*
Treatment from the family doctor is in line with treatment from other care providers	84.7*
Emotional support	
Emotional support was also provided	53.0
Attention was paid to possible feelings of fear, gloom, and anxiety	54.5
I was made aware of the possibilities for more intensive emotional support	32.4
Attention was paid to the impact of my health on my private life (family, relatives, work, social life)	52.0
Access to care	
It was no problem to go from my home to my family doctor and back again	80.4
The general practice was easily accessible	94.7
I could easily schedule an appointment quickly	85.6
On a visit I didn’t have to wait long before it was my turn	69.4
I could easily request a prescription refill	93.3
Information and education	
I was well informed	87.5
The information I received was well explained	85.1
I had easy access to my own data (lab results, medication overview, referrals)	55.2
I could ask all the questions I wanted	89.6
Family and friends	
With my consent, relatives were involved in my treatment	70.5*
Attention was given to care and support provided by family members	57.4*
Attention was given to possible questions from my family members	63.3*

Note: Based on non-imputed data. *If applicable

The results of the correlation analysis are displayed in Table 3. PCC and co-creation of care were correlated significantly with patients’ physical well-being, social well-being, and satisfaction with care (all *p* ≤ 0.001). In addition, a weak negative correlation was found between satisfaction with care and single marital status (*r* = − 0.148, *p* = 0.033). Physical well-being was correlated negatively with age (*r* = − 0.165, *p* = 0.016). A weak positive correlation was found between physical well-being and male gender (*r* = 0.152, *p* = 0.029). All eight dimensions of PCC were correlated significantly with patients’ physical well-being, social well-being, and satisfaction with care (Table 4). Finally, a positive relationship was found between PCC and co-creation of care (*r* = 0.442, *p* < 0.001).

**Table 3:** Associations between patients’ characteristics, patient-centered care, co-creation of care and satisfaction and social and physical well-being (*n* = 216)

	Satisfaction with care		Social well-being		Physical well-being	
Variable	*r*	*p*	*r*	*p*	*r*	*p*
Age (years)	−0.121	0.080	−0.006	0.927	−0.165	0.016
Gender (male)	0.110	0.155	0.057	0.407	0.152	0.029
Marital status (single)	−0.148	0.033	−0.011	0.870	−0.129	0.064
Education (low)	−0.080	0.263	−0.050	0.473	−0.131	0.064
Patient-centered care	0.501	< 0.001	0.446	< 0.001	0.392	< 0.001
Co-creation of care	0.389	< 0.001	0.334	< 0.001	0.217	0.001

Note: Based on imputed data

**Table 4:** Relationships of the eight patient-centered care dimensions and co-creation of care with satisfaction and social and physical well-being (*n* = 216)

PCC dimension or co-creation of care	Satisfaction with care		Social well-being		Physical well-being	
	*r*	*p*	*r*	*p*	*r*	*p*
Patients’ preferences	0.446	< 0.001	0.324	< 0.001	0.333	< 0.001
Physical comfort	0.371	< 0.001	0.367	< 0.001	0.325	< 0.001
Coordination of care	0.475	< 0.001	0.363	< 0.001	0.294	< 0.001
Emotional support	0.309	< 0.001	0.307	< 0.001	0.183	0.011
Access to care	0.454	< 0.001	0.324	< 0.001	0.333	< 0.001
Continuity and transition	0.442	< 0.001	0.335	< 0.001	0.203	0.010
Information and education	0.416	< 0.001	0.398	< 0.001	0.280	< 0.001
Family and friends	0.308	< 0.001	0.332	< 0.001	0.202	0.013
Overall PCC	0.501	< 0.001	0.446	< 0.001	0.329	< 0.001
Co-creation of care	0.389	< 0.001	0.334	< 0.001	0.217	< 0.001

Note: Based on imputed data. PCC, patient-centered care

The results of the multivariate regression analyses are presented in Table 5. After controlling for background characteristics, PCC was associated with social well-being (*B* = 0.387, *p* ≤ 0.001), physical well-being (*B* = 0.368, *p* ≤ 0.001), and satisfaction with care (*B* = 0.425, *p* ≤ 0.001). Co-creation of care was associated with social well-being (*B* = 0.112, *p* = 0.006) and satisfaction with care (*B* = 0.119, *p* = 0.007). Although we found a significant association between co-creation of care and physical well-being in the bivariate analysis, this effect dissipated in the multivariate analysis (*B* = 0.062, *p* = 0.249). The significant associations of background characteristics with satisfaction with care and physical well-being also dissipated in the multivariate analysis.

**Table 5:** Multivariate relationships of variables with satisfaction with care, social well-being, and physical well-being (*n* = 216)

Variable	Satisfaction with care	Social well-being			Physical well-being	
	*B* (SE)	*p*	*B* (SE)	*p*	*B* (SE)	*p*
Age	0.004 (0.003)	0.210	0.000 (0.003)	0.932	−0.005 (0.004)	0.241
Gender	0.019 (0.059)	0.785	0.018 (0.072)	0.770	0.107 (0.086)	0.210
Marital status	−0.055 (0.070)	0.648	0.051 (0.062)	0.233	−0.038 (0.088)	0.667
Education	−0.068 (0.080)	0.397	−0.061 (0.062)	0.326	−0.099 (0.095)	0.297
Patient-centered care	0.425 (0.078)	≤0.001	0.387 (0.069)	≤0.001	0.368 (0.097)	≤0.001
Co-creation of care	0.119 (0.044)	0.007	0.112 (0.039)	0.006	0.062 (0.054)	0.249

Note: Based on imputed data

Adjusted R^2^ social well-being: 0.18

Adjusted R^2^ physical well-being: 0.11

Adjusted R^2^ satisfaction with care: 0.31

## Discussion

This study demonstrated that the eight dimensions of PCC and co-creation of care are important for satisfaction with care, physical well-being, and social well-being among patients with multi-morbidity in the primary care setting in Noord-Brabant, the Netherlands. Although similar findings have been obtained among patients in hospital settings [11] and for care delivery to people with intellectual disabilities [13], this study is the first to show the importance of both PCC and co-creation of care for patients with multi-morbidity in the primary care setting. This patient population experiences lower levels of social and physical well-being than do patients with single chronic diseases, such as COPD, CVD, and diabetes [29–31]. Patients with multi-morbidity differ in many other aspects from patients with single chronic diseases. Hopman, Schellevis, and Rijken [32] showed that patients with multi-morbidity are more often male and less educated, and that they experience more problems in health domains such as mobility, usual activities, and pain/discomfort. Thus, care needs to be made more patient-centered and tailored to the needs of patients with multi-morbidity.

Although the overall level of PCC in the primary care practices included in this study was sufficient, there is room for improvement in two dimensions in particular: family and friends, and emotional support. More than one-quarter of all patients with multi-morbidity in this study were not completely satisfied with aspects of the involvement of family and friends in their care. Moreover, this dimension was not considered to be applicable for almost half of the study population; 43% of patients were single, which could reflect an absence of family members who could be involved in the care process. Chronically ill patients who are married or have partners are more likely to bring these partners to GP visits [33]. Furthermore, previous studies have shown that two-thirds of care providers endorse barriers to the participation of family and friends in patients’ care processes; they are concerned about privacy rules, they experience the involvement of family and friends as burdensome, and/or they are uncertain about their skills for such involvement [33].

About half of the patients surveyed in this study did not experience sufficient levels of emotional support from their care providers. Kenning and colleagues [34] revealed a discrepancy between the expectations and experiences of patients with multi-morbidity and their care providers in the primary care setting. Further research should focus on how emotional support should be provided to meet patients’ needs.

In the bivariate analyses, co-creation of care was related positively to satisfaction with care, physical well-being, and social well-being. However, the effect of physical well-being dissipated in the multivariate analyses. The stronger association between co-creation of care and social well-being could be explained by the fact that the former focuses mainly on social aspects, namely the quality of a relationship [14]. The key elements of co-creation of care (shared goals, shared knowledge, mutual respect) enable the realization of social well-being goals. To illustrate, mutual respect between patients and care providers may result in higher levels of status for patients, as when they receive compliments from care providers on how they are dealing with their conditions relative to other patients or compared to how they used to deal with their conditions. Acknowledging a patient’s specific care needs may result in more affectionate and trusting interactions with the care provider, fulfilling the patient’s need for affection and behavioral confirmation. Co-creation of care may add to social well-being through the quality of patient-centered interaction and communication. However, when a patient’s physical health deteriorates, this quality is unlikely to improve or change his/her physical status. Currently, most researchers do not consider physical and social well-being separately; rather, they combine the concepts into a single overall well-being or quality of life score. The findings of this study demonstrate the importance of separately examining physical and social well-being in future research on PCC and co-creation of care.

This study has several limitations that should be taken into account when interpreting our findings. First, the cross-sectional design prevented us from determining the causality of relationships. Second, this study was conducted in Noord-Brabant, a region in the Netherlands; research in other regions and/or countries is needed to confirm our study findings. Third, this study assessed the experiences of patients with multi-morbidity, which does not guarantee the objectivity of observations and measurements; however, subjective experiences and self-rated health are important predictors of health outcomes, such as morbidity and mortality [35]. The final limitation is the response rate. Although the response rate of 55% might be considered as low, it is higher compared to other studies in which the respondents also received a questionnaire by mail [36, 37] and much higher compared to earlier studies using the same strategy among chronically ill patients (31% response rate) [38]. Our sample still may be biased which could have affected our study findings; non-responders may have been in poorer health compared to those who did fill in the questionnaire.

## Conclusion

PCC and co-creation of care are associated positively with satisfaction with care and the physical and social well-being of patients with multi-morbidity in the primary care setting. These findings are important because current care delivery is not tailored to the needs of patients with multi-morbidity, although multi-morbidity is often related to adverse patient outcomes. Making care more tailored to the needs of these patients by paying attention to PCC and co-creation of care may contribute to better outcomes.

## Additional file

Additional file 1: **Table S1** Descriptive statistics of physical and social well-being in patient populations with multi-morbidity, COPD, CVRM, and diabetes. (DOCX 16 kb)

## Abbreviations

COPD: Chronic obstructive pulmonary disease
CVD: Cardiovascular disease
GP: General practitioner
PCC: Patient-centered care
PCPC: Patient-centered primary care
SASC: Satisfaction with Stroke Care
SD: Standard deviation
SPF-ILs: Social Production Function Instrument for the Level of Well-being
