# Supplementary material for: The importance of patient-centered care and co-creation of care for satisfaction with care and physical and social well-being of patients with multi-morbidity in the primary care setting
Source: BMC Health Serv Res. 2019 Jan 8;19:13. doi: 10.1186/s12913-018-3818-y (PMC6323728; doi:10.1186/s12913-018-3818-y)
Supplement: Supplementary file 1 — Table S1 Descriptive statistics of physical and social well-being in patient populations with multi-morbidity, COPD, CVRM, and diabetes. (DOCX 16 kb) [file 12913_2018_3818_MOESM1_ESM.docx]

**Additional file 1**

| **Table S1** Descriptive statistics of physical and social well-being in patient populations with multi-morbidity, COPD, CVRM, and diabetes | | | | | | | | |
| --- | --- | --- | --- | --- | --- | --- | --- | --- |
|  | Multi-morbidity |  | COPD |  | CVRM |  | Diabetes |  |
| Statistic | Physical well-being | Social well-being | Physical well-being | Social well-being | Physical well-being | Social well-being | Physical well-being | Social well-being |
| *n* | 216 | 216 | 400 | 390 | 439 | 439 | 135 | 134 |
| Mean ± standard deviation (range) | 2.55 ± 0.62 (1–4) | 2.71 ± 0.53 (1.44–4) | 2.76 ± 0.58 (1–4) | 2.81 ± 0.55 (1–4) | 2.78 ± 0.52 (1.5–4) | 2.79 ± 0.49 (1–4) | 2.79 ± 0.49 (1–4) | 2.82 ± 0.47 (1.33–4) |
| Note: Well-being data for patients with COPD (Chronic Obstructive Pulmonary Disease), CVRM (Cardio Vascular Risk Management), and diabetes are derived from 2012 surveys of chronically ill patients enrolled in Dutch disease management programs; the 15-item version of the Social Production Function Instrument for the Level of Well-being short version was used to assess physical and social well-being. This survey was part of a larger study of the effectiveness of disease management programs in the Netherlands [Lemmens KMM, Rutten-Van Mölken MPMH, Cramm JM, Huijsman R, Bal RA, Nieboer AP. Evaluation of a large scale implementation of disease management programmes in various Dutch regions: a study protocol. BMC Health Serv Res. 2011;11:6.]. | | | | | | | | |
